# Evaluating Maize Hybrids for Yield, Stress Tolerance, and Carotenoid Content: Insights into Breeding for Climate Resilience

**DOI:** 10.3390/plants14010138

**Published:** 2025-01-06

**Authors:** Călin Popa, Roxana Elena Călugăr, Andrei Varga, Edward Muntean, Ioan Băcilă, Carmen Daniela Vana, Ionuț Racz, Nicolae Tritean, Ioana Virginia Berindean, Andreea D. Ona, Leon Muntean

**Affiliations:** 1 Department of Plant Breeding, Faculty of Agriculture, University of Agricultural Sciences and Veterinary Medicine Cluj-Napoca, 3-5 Mănăstur St., 400372 Cluj-Napoca, Romania; calin-vasile.popa@student.usamvcluj.ro (C.P.); andreea.ona@usamvcluj.ro (A.D.O.); leon.muntean@usamvcluj.ro (L.M.); 2 Agricultural Research and Development Station Turda, Agriculturii 27, 401100 Turda, Romania; andrei_varga06@yahoo.com (A.V.); emuntean@usamvcluj.ro (E.M.); carmend.vana@yahoo.com (C.D.V.); ionut.racz@usamvcluj.ro (I.R.); nicu.tritean@scdaturda.ro (N.T.); 3 Food Sciences Department, Faculty of Food Sciences and Technology, University of Agricultural Sciences and Veterinary Medicine Cluj-Napoca, 3-5 Mănăstur St., 400372 Cluj-Napoca, Romania; 4 Institute of Biological Research, Branch of the National Institute of Research and Development for Biological Sciences, Department of Experimental Biology and Biochemistry, 48 Republicii Street, 400015 Cluj-Napoca, Romania; ioan.bacila@icbcluj.ro; 5 Department of Genetics, Faculty of Agriculture, University of Agricultural Sciences and Veterinary Medicine Cluj-Napoca, 3-5 Mănăstur St., 400372 Cluj-Napoca, Romania

**Keywords:** general combining ability, special combining ability, total carotenoids, zeaxanthin, lutein, yield, heterosis

## Abstract

To ensure food and feed security, modern maize hybrids must not only perform well under changing climate conditions but also consistently achieve higher and stable yields, exhibit maximum tolerance to stress factors, and produce high quality grains. In a study conducted in 2022 and 2023, 50 maize hybrids were developed from crosses of five elite (highly productive) inbred lines and ten lines possessing favorable genes for carotenoid content. These hybrids were tested under particularly unfavorable conditions for maize cultivation. The aim was to identify which lines effectively transmit the desired traits to the offspring (general combining ability—GCA), and to identify superior hybrids in terms of productivity, adaptability, and quality (specific combining ability—SCA). The study revealed that total carotenoids ranged from 2.30 to 40.20 μg/g for the inbred lines and from 7.45 to 25.08 μg/g for hybrids. A wider distribution of values was observed in the inbred lines compared to the hybrids for key carotenoids such as lutein, zeaxanthin, β-cryptoxanthin, and β-carotene. Among the hybrids, notable performers in yield, adaptability, and carotenoid content included E390×D302, A452×D302, and A447×D302. The paternal inbred line D302 exhibited a high general combining ability for yield (1446 kg ha^−1^) and, when crossed with several inbred lines, produced hybrids with enhanced yields and higher levels of zeaxanthin, lutein, and β-carotene, as well as improved unbroken plants percent.

## 1. Introduction

Continuous population growth has driven breeders to create superior genotypes with enhanced productivity and quality; given the scale of cultivation and production, maize yield improvement, adaptation to stress factors, and achieving superior grain quality have become key objectives. In Romania, maize is the crop that occupy the largest cultivated area, covering approximately 2.5 million hectares annually [1]. Although in the past national researches focused on maize carotenoid content, these were deprioritized as breeders concentrated on maximizing production. However, recent years have seen a resurgence on interest in improving the grain quality, particularly by increasing the carotenoid content [2,3,4,5].

Improving the biochemical content of maize grains has garnered significant attention among researchers, with diverse objectives being pursued. Key directions include enhancing starch content by altering the amylopectin [6] or amylose [7,8] proportions, and improving sugar content for sweet corn varieties [9,10,11,12]. Another area of focus is maize oil, which is an important source of tocopherols [13], driving interest in enriching this component. Protein improvement is also a priority, targeting both quantity and quality [14,15,16,17], with efforts to increase lysine and tryptophan [18,19,20,21] and reduce zein content [22]. Biofortification of maize grains with essential micronutrients such as iron [23,24], carotenoids [25,26,27,28], and vitamins [21,29,30] has emerged as a critical research domain. Although maize grains have lower carotenoid concentrations compared to certain fruits or vegetables, their importance lies in their high global consumption.

The increasing focus on carotenoid content stems from their significant human health benefits. Studies have demonstrated that a carotenoid-rich diet positively impacts health by reducing the risk of cardiovascular diseases [31,32,33,34], obesity [35], certain cancers [36,37], and diabetes [38]. Carotenoids also enhance the immune system [39,40], improve eye health [41,42,43] by protecting the retina from damaging blue light (lutein and zeaxanthin), and offer antioxidant benefits [44,45].

Maize grains contain a relatively wide range of carotenoids, the major ones being lutein, zeaxanthin, β-cryptoxanthin, α-carotene, and β-carotene, with lutein and zeaxanthin being predominant. Their concentration varies a lot, depending on the analyzed genotypes: reported values for zeaxanthin range from 0 to 43.9 μg/g [29,46,47], while lutein ranges from 0 to 53 μg/g [47,48]. β-cryptoxanthin and β-carotene levels range from 0 to 13,8 μg/g and 0 to 13.6 μg/g, respectively [46,48]. Similar values have been reported for inbred lines and hybrids created at the Agricultural Research and Development Station (ARDS) Turda [2,3].

The importance of maize for our country is obvious: Romania is the largest maize growing country from the UE, in terms of harvested area. According to FAOSTAT data [1], in recent years the areas cultivated were constant, between 2.3 and 2.6 million hectares, but the total production varied, between 8 and 18.6 million tons. The extreme drought reported in several parts of the country resulted in a very low yield or even a total compromise in some areas in 2022, thus total production was very low (8 million tons).

Developing nutritionally superior maize hybrids capable of thriving under changing climate conditions has become a priority for ensuring food and feed security. In recent years, Europe and Romania have experienced extreme heat and water deficits, adversely impacting maize production [49]. Therefore, breeders are now tasked with achieving stable, high-yielding genotypes that are resilient to stress factors while also offering superior grain quality.

Good results regarding drought improvement were obtained at NARDI Fundulea, in the southern part of Romania, an area subject to thermal and hydric stress in recent years. However, due to the different pedoclimatic conditions, the maize hybrids obtained in the south are recommended for areas where they can be sown earlier, and their vegetation period is longer (FAO > 350), thus implicitly their yield capacity is different [50,51,52]. In the western part of Romania, at ARDS Lovrin, breeding for tolerance to extreme temperatures is also carried out [53,54,55], but again this area has a different specific climate, the hybrids being recommended for more favorable areas. Transylvania (Central) and Moldova (East) are not as favorable to maize cultivation as the other areas mentioned; here, early sowing is not recommended due to increased risk of low spring temperatures, while early frosts are also encountered, so genotypes with a longer vegetation period are not recommended in these areas. For these areas, FAO 300–380 hybrids are recommended, while FAO 200–300 hybrids are recommended in higher areas.

In Romania there are few areas where agricultural crops are irrigated, about 10% of the country, although half of the country is subject to drought conditions [56]. Most of the irrigated areas are in the south and southeast of the country, being very rarely found in Transylvania or Moldova. Although some programs aim to increase the agricultural areas with the possibility of irrigation, until then, breeders must create hybrids that can be productive even in dry conditions.

In this context, 50 potentially superior maize hybrids were tested at ARDS Turda. Their yields and their carotenoid content were monitored in two years of unfavorable conditions for maize cultivation: 2022 and 2023. The objectives of the study were: (1) to identify parental inbred lines possessing the favorable crtRB1 and lcyE genes, capable of transmitting enhanced carotenoid content to hybrids; and (2) to identify hybrids exhibiting a high content in carotenoids alongside increased production capacity and robust tolerance to heat and water stress.

## 2. Results

### 2.1. Carotenoid Content in Parental Inbred Lines

The per se values for total carotenoids in the maternal inbred lines ranged from 10.64 to 23.44 μg/g, while those in paternal lines varied more widely, from 2.30 to 40.20 μg/g (Table 1). The high total carotenoid content of the paternal line D302 (40.20 μg/g) was associated with the highest content in β-cryptoxanthin (6.43 μg/g) and β-carotene (3.22 μg/g). The line that stood out for the highest content in lutein is N4 (19.68 μg/g), and LC62 for zeaxanthin (15.74 μg/g). These findings highlight the potential of specific inbred lines for breeding hybrids with enhanced carotenoid profiles.

**Figure 1:**
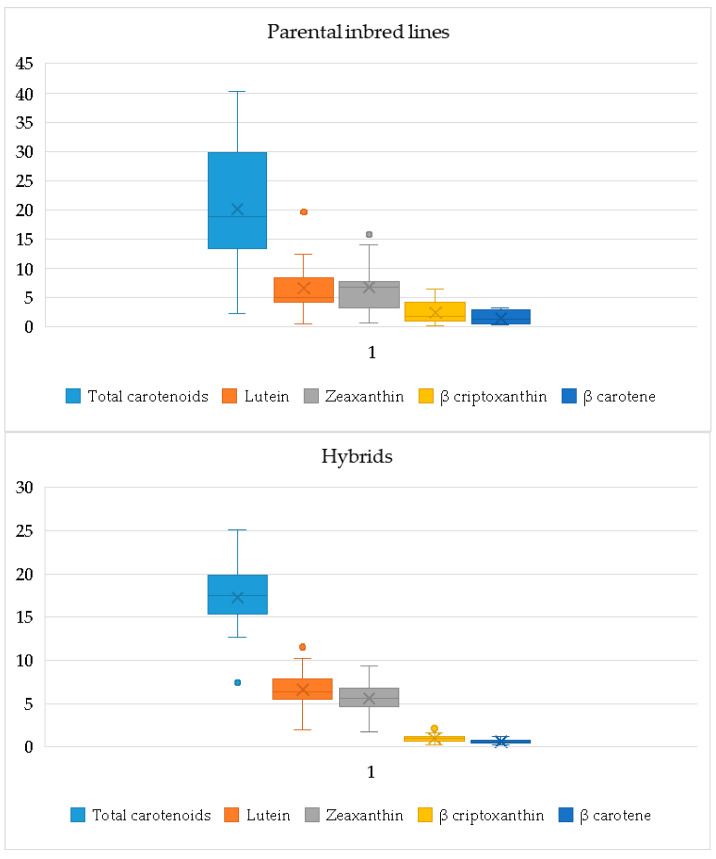
Distribution of carotenoid levels (μg/g) in parental inbred lines and hybrids.

The distribution of carotenoid content (Figure 1) was wider in the case of the inbred parental forms compared to the hybrids resulting from their crosses. Total carotenoid levels had values between 2.3 and 40.2 μg/g for the inbred lines, whereas the hybrids exhibited a narrower range of 7.45 to 25.08 μg/g. Lutein varied between 0.46 and 19.68 μg/g for inbred lines and between 1.95 and 11.56 μg/g for hybrids, while for zeaxanthin the values ranged between 0.69 and 15.74 μg/g (inbred lines) and 1.71 and 9.34 μg/g (hybrids). For β-cryptoxanthin and β-carotene, the values ranged between 0.14–6.43 μg/g and 0.35–3.22 μg/g (inbred lines), respectively, while the hybrids showed narrower range, of 0.21–2.1 μg/g and 0.22–1.24 μg/g, respectively.

The average concentrations in the inbred lines were 20.14 μg/g for total carotenoids, 6.63 μg/g for lutein, 6.76 μg/g for zeaxanthin, 2.46 μg/g for β-cryptoxanthin, and 1.52 μg/g for β-carotene, whereas the hybrids had slightly lower average values of 17.29 μg/g for total carotenoids, 6.64 μg/g for lutein, 5.58 μg/g for zeaxanthin, 1.0 μg/g for β-cryptoxanthin, and 0.60 μg/g for β-carotene.

### 2.2. General Combining Ability (GCA) of the Maternal Inbred

The additive effects of maternal lines indicate line E390 as favorable for achieving higher zeaxanthin content (Table 2). Line A452 is notable for transmitting both higher yield and enhanced lutein content. Line A483 demonstrates strong GCA for fat content, while E385 contributes to earliness (reflected in higher dry matter content at harvest) and a greater percentage of zeaxanthin, but earliness is associated with lower grain yield.

### 2.3. GCA of the Paternal Inbred Lines

Analysis of the GCA effects of the paternal inbred lines (Table 3) reveals that D302 contributes significantly at the additive level to increased yield, along with a favorable influence on total carotenoids, lutein, and zeaxanthin. Another standout is line N4, which exerts a positive additive effect on total carotenoids and lutein while also significantly enhancing protein content. The LC62 line shows favorable effects across four traits: dry matter, protein, total carotenoids, and zeaxanthin. Additionally, the paternal line C382 had favorable effects on zeaxanthin and β-cryptoxanthin content.

### 2.4. Agronomic Performance of the Studied Hybrids

Both 2022 and 2023 were among the least favorable years for maize production in Romania and the studied area [49], due to thermal and water stress during the vegetation period: in the summer months, temperatures exceeding the area’s specific values were recorded, associated with a lack of precipitation. Despite these challenging condition, superior genotypes were identified, demonstrating strong tolerance to drought and heat.

Several hybrids stood out for their productive superiority, indicating good adaptability to the unfavorable conditions of the experimental years: E390×D302, A452×D302, A447×D302, A452×LPN, and A483×D302. The influence of the favorable interaction between the D302 line and the elite lines for grain yield is obvious (Table 4). These hybrids significantly exceeded the experimental average yield (7119 kg ha^−1^) at statistical thresholds of *p* < 0.05 = 650, *p* < 0.01 = 858, and *p* < 0.001 = 1105, with differences reaching up to 2281 kg ha^−1^.

The three most productive hybrids also displayed a good synchronization between flowering and stigma appearance (low ASI), thus favoring pollination. ASI values varied across the hybrids, ranging from −2 to 7. Notably, two hybrids had an ASI of −2, three had a value of −1 (one of which was also the most productive in the entire experimental system), and thirteen hybrids demonstrated coincidence between flowering and stigma appearance (BBCH 63–65).

C382, a line that stood out for its good GCA regarding the percentage of unbroken plants, also exhibited excellent special combining ability (SCA) in crosses with the elite lines A474 (100%), A483 (99.3%), and E390 (98.6%) at significance thresholds of *p* < 0.05 = 6.8, *p* < 0.01 = 9.0, and *p* < 0.001 = 11.6.

Regarding the dry matter percentage, the maternal elite line E385 has a notable influence, transmitting precocity to its hybrids; consequently, the earliest seven hybrids across the experimental system shared this line as their parental genotype, with statistical significance at *p* < 0.05 = 1.3, *p* < 0.01 = 1.7, and *p* < 0.001 = 2.2.

**Figure 2:**
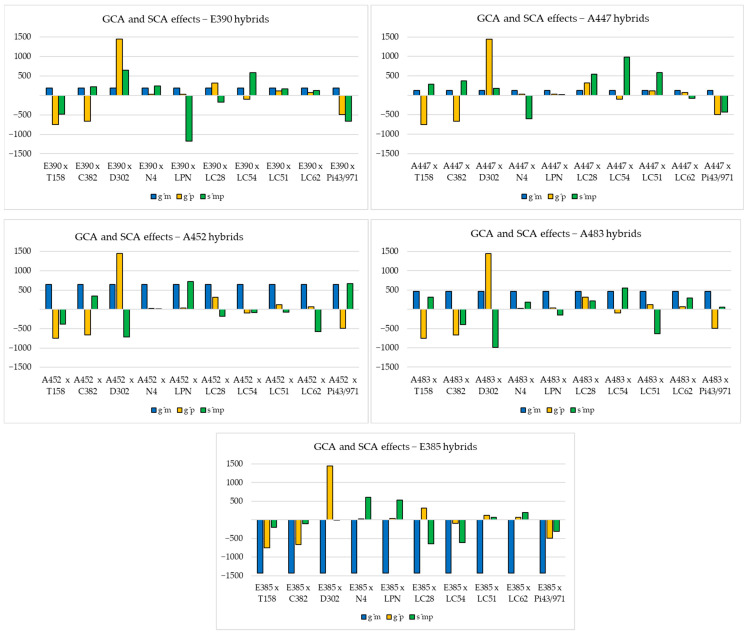
GCA effects of parental inbred lines and SCA effects of studied hybrids, for yield.

Following the analysis of the GCA and SCA effects (Figure 2), it is possible to observe the clear favorable influence of some parental lines in achieving the yield (A452, A483, D302), while in the case of some hybrids, it was mainly influenced by the interaction between the parental forms. In the case of hybrids from the maternal line group E385, the unfavorable influence of the line is evident.

### 2.5. Grain Quality in the Studied Hybrids

The protein content varied between 7.58 and 10.52% (mean value of 8.85%), and the following hybrids exhibiting the highest content: E385×LC28, E390×LC57, A483×LC62, and E385×C382, at significance thresholds of *p* < 0.05 = 0.28, *p* < 0.01 = 0.37, and *p* < 0.001 = 0.48 (Table 5).

The influence of the maternal line A483 on fat content, previously highlighted in GCA analysis, was also evident in crosses with lines LC28 (4.93%), LC54 (4.45%), T158 (4.30%), and LC57 (4.25%), with statistical significance thresholds of *p* < 0.05 = 0.57, *p* < 0.01 = 0.76, and *p* < 0.001 = 1.0.

In terms of fiber content, the hybrids A483×LPN, A483×T158, E390×LC62, and A447×LC62 stood out for exceeding the mean value (2.35%), with significance levels of *p* < 0.05 = 0.37, *p* < 0.01 = 0.49, and *p* < 0.001 = 0.64.

Certain hybrids combined higher levels of protein, fat, and fiber, including A483×T 158 (9.63/4.30/2.94), A483×LC28 (9.16/4.93/2.82), and A483×LC62 (9.68/3.54/2.24).

### 2.6. Carotenoid Content of the Studied Hybrids

In terms of total content of carotenoids (Table 6), the following hybrids exhibited statistically significant higher values, compared to the experimental average of 17.29 μg/g: E390×LC62 (+7.79 μg/g), A452×D302 (+5.03 μg/g), A447×D302 (+4.56 μg/g), A483×T158 (+4.22 μg/g), and A452×N4 (+3.46 μg/g), with significance levels of *p* < 0.05 = 1.61, *p* < 0.01 = 2.15, and *p* < 0.001 = 2.80. For lutein content, the following were notable: A452×N4, A447×D302, A447×N4, and A452×D302 (*p* < 0.05 = 0.72, *p* < 0.01 = 0.96, *p* < 0.001 = 1.25). Hybrids A447×D302 and A452×D302 also stood out for their high productivity in the entire experimental system and demonstrated a very good coincidence at flowering.

The highest zeaxanthin content was observed in hybrids E390×LC62, E385×LC68, E385×LC51, and A483×T158 (*p* < 0.05 = 0.50, *p* < 0.01 = 0.66, *p* < 0.001 = 0.87). For β-cryptoxanthin, the hybrids A483×C382, E390×C382, A483×T158, and A452×C382 were noted (*p* < 0.05 = 0.32, *p* < 0.01 = 0.42, *p* < 0.001 = 0.55). For β-carotene, the highest content was found in hybrids E390×LC57, E390×N4, E385×N4, and A483×T158 (*p* < 0.05 = 0.22, *p* < 0.01 = 0.30, *p* < 0.001 = 0.39).

The A483×T158 hybrid, which also stood out for its high protein, fat, and fiber content, demonstrated a favorable performance in terms of SCA for total carotenoids (21.51 μg/g), zeaxanthin (7.55 μg/g), cryptoxanthin (1.59 μg/g), and beta-carotene (0.94 μg/g), statistically significantly exceeding the experimental average. Crosses between the maternal lines A447 and A452 with the paternal line D302 stood out for their above average content of total carotenoids and lutein. Additionally, E390×LC62 stood out due to high content of both total carotenoids and zeaxanthin.

### 2.7. MPH% for Carotenoid Content

The largest number of hybrids exhibiting positive mpH% heterosis (Table 7) was observed for lutein (29 hybrids) followed by total carotenoids (17 hybrids) and zeaxanthin (16 hybrids). The mid parent heterosis reached up to 99.3% for lutein and 68.7% for β-cryptoxanthin. Hybrid A483×LPN exhibited mpH% for all carotenoids, being also characterized by a higher fiber content and a high percentage of unbroken plants; however, in terms of yield, it had values close to the average. Several hybrids were noted for positive mpH% for total carotenoids, lutein, and zeaxanthin, including: E390×LC54, E390×LC57, A452×T158, A452×C382, A452×LC54, A452×LC57, A452×Pi43-971, A483×T158, E385×LC54, and E385×LC57.

Some hybrids exhibited negative MPH%, which can be explained by the fact that both parental inbred lines had among the highest values for carotenoids per se: E385×D302, E385×LC28, E385×LC62, E385×Pi43/971, A483×D302, A483×LC28, and A483×LC62.

## 3. Discussion

Carotenoids represent a group of phytochemicals responsible for several colors in plants [57]. They are among the most common natural pigments, with over 850 different compounds identified and characterized [58,59]. α-carotene, β-carotene, and β-cryptoxanthin are the major vitamin A precursors, while the non-provitamin A carotenoids are lycopene, lutein, and zeaxanthin [59,60]. These compounds are synthesized by plants and microorganism, but not by animals, thus it is necessary for people to ensure their needs by consuming fruits and vegetables with a high content of carotenoids. Vitamin A deficiency is one of the most common problems among human populations and since it cannot be synthesized in human body, it needs to be supplied externally either as supplement or through consumption of balanced diet [61]. Vitamin A is required for proper cell growth, eye vision, immune functions, and reproductive system [62,63,64]. Consumption of carotenoids is also associated with reduced risks of some cancers and other serious conditions (diabetes, obesity, cardiovascular disease) while also improving skin health [58,65].

The level of carotenoids in various fruits and vegetables is higher than in maize, but it retains its importance due to its high consumption and multiple uses in human nutrition, as well as its accessibility and possibility of consumption in areas with a lower economic index [66]. Maize can be a carotenoid source for humans and animals as it usually contains lutein and zeaxanthin as major pigments [67].

Although there are genetic methods to increase the carotenoid content of maize, in some regions, classical breeding methods are preferred. Thus, to improve the composition of the grains, backcrosses can be performed with genotypes for which a higher carotenoid content has been quantified. Lines with high carotenoid content can also be used in crosses to obtain new maize hybrids [47]. Marker-assisted selection is also of particular importance, the identification of favorable genes facilitating field work, reducing the samples needed to be quantified using HPLC. This allows the identification of genotypes that can be used in classical recurrent selection programs [18,68,69,70]. The crtRB1 and lcyE allele selection could be successfully used in the marker-assisted selection, saving significant time and cost.

Two genes are important in the accumulations of provitamin A carotenoids in maize kernel: crtRB1 and lcyE. The crtRB1 gene is mapped on chromosome 10 and encodes β-carotene hydroxylase enzyme. The strong and significant effect of the crtRB1-favourable allele for enhanced β-carotene in maize is very well established [2,69,70,71,72]. The lcyE gene, mapped to chromosome 8, consists of ten exons and modifies the downstream α-carotene to β-carotene branches of the carotenoid biosynthesis pathway [46]. Several studies have highlighted the importance of crtRB1 and lcyE interaction genes for provitamin A accumulation in maize [70,72,73,74,75]. Alleles of both genes have a low frequency in maize germplasm, so marker-assisted pyramiding could be used to combine them in a certain genotype [76].

GCA analysis is important in maize breeding due to the possibility of identifying parental inbred lines that transmit certain traits of interest and establishing effective breeding strategies. GCA testing requires a large number of crossings, with common testers, in factorial or diallel system, depending on the genetic base and the number of lines and tests, as well as according to the purpose followed [77]. Maize germplasm is very varied, regarding the vegetation period, production capacity, adaptability, grain biochemical composition, vegetative traits, etc., so to increase the chances of obtaining a superior hybrid, it is necessary to test a line with several tester lines.

The combining ability of fifteen inbred lines (five maternal and ten paternal) for carotenoid and important agronomic traits was investigated. 50 hybrid combinations were tested, resulting from crossing five elite inbred lines and ten lines possessing one or both genes favoring carotenoids. Since several favorable traits must be combined to obtain a competitive maize hybrid, the following were considered: yield, % of erect plants at harvest, dry matter, ASI (as an indicator of drought tolerance during the anthesis period), and the biochemical composition of the grains (protein, fat, fiber), as well as total carotenoids, lutein, zeaxanthin, β-cryptoxanthin, and β-carotene. The aim was both to identify the lines that transmit as many traits as possible (GCA), so that they can be used for the creation of new hybrids or in selection programs, as well as to identify hybrids that combine several favorable traits (SCA) and could be promoted further. The inbred lines used are different in agronomic and vegetative traits, grain composition, and reaction to stress factors, so a great diversity of hybrids was expected. An increased variability was observed for all traits of the hybrids, these being influenced both by the parental lines and by the interaction between the two.

Significant GCA effects for carotenoid content indicate their control by additive gene effects, while significant SCA effects indicate the implication of non-additive gene effect, a conclusion also stated by other authors [78]. Thus, the following inbred lines were identified which, due to their GCA, can be used as parental forms or as sources for the improvement of other lines: D302, N4, LC62, and C382.

Both parental inbred lines and the 50 resulting hybrids showed broad ranges of variation for the studied carotenoids. The distribution of carotenoid levels in the parental inbred lines and hybrids shows a broader range for the inbred lines for all analyzed components. The range for total carotenoid was 2.3–40.20 μg/g for inbred lines and 7.49–25.08 μg/g for hybrids. The lutein range was 0.46–19.68 μg/g for inbred lines and 1.95–11.56 μg/g for hybrids. For zeaxanthin, the range was 0.69–15.74 μg/g in inbred lines and 1.71–9.34 μg/g in hybrids. The β-cryptoxanthin content reached up to 4.43 μg/g in inbred lines and 2.10 μg/g in hybrids, while the maximum values for β-carotene were 3.22 μg/g in inbred lines and 1.24 μg/g in hybrids. These values are consistent with those reported by other researchers [25,29,47,62,78].

The highest total carotenoids and zeaxanthin values were observed in the hybrid E390×LC62, which also showed a high SCA for these components. This hybrid also had good yield under unfavorable conditions, close to the experimental average. The highest yield in the experimental system was obtained by E390×D302, a hybrid that also had a low ASI (−1 and −2) under heat stress conditions and high carotenoid content. The hybrid noted for the most favorable traits was A447×D302 (Figure S1; it achieved an average yield of 8490 kg ha^−1^, exhibited good flowering–silking coincidence, and had total carotenoids of 21.85 μg/g and 10.22 μg/g lutein. It also performed well in the dry conditions of the experimental years, and is being tested in 2024, a year expected to be similarly unfavorable. Due to its ability to stay green for longer periods under stress, low ASI, good yield, good grain quality, this hybrid is proposed for registration and cultivation in the area. During the experimentation for registration, this hybrid will be tested in several areas of the country, including with drier or colder conditions, and will be registered and marketed only if in at least two years it surpasses the controls.

In the area where the experiment was carried out, 2022 and 2023 were among the least favorable years for maize cultivation [49], but in breeding programs, such years allow the selection of the most tolerant genotypes. The effects of stress factors must be followed both in the case of hybrids and in the parental forms. Some older genotypes (such as the paternal lines in our case) have not benefited from a longer period of improvement of the genetic basis [79], and may be more sensitive to some factors, such as climatic conditions. In the seed production for maize hybrids, greater attention must be paid to the behavior of the parental forms, and only the superior productive and adaptive ones must be used. For example, if a sensitive line is used, in years with dry conditions, symptoms such as the drying of the panicle and the leaves, increased anthesis–silking interval, and poor pollination can occur [80,81,82], so that the hybridization batches are compromised. This risk is present especially in areas where irrigation is not carried out, such as Transylvania. The sensitivity can be transmitted to the hybrids, resulting in significant losses for farmers. That is precisely why it is necessary to test the genotypes over several years, under different conditions, in order to have as broad a picture of their behavior as possible.

## 4. Materials and Methods

### 4.1. Biological Material

Out of the desire to increase the production of the maize crop, for many years the quality of the grains had less importance, but due to the importance it presents, at ARDS Turda we resumed and intensified the research on improving the quality, especially the carotenoid content. As a first step, a molecular screening was performed to identify the inbred lines possessing one or both favorable alleles of crtRB1 and lcyE genes [2]. A total of 2746 inbred lines from Romania were tested, and 748 of these belong to the ARDS Turda germplasm collection.

DNA isolation and PCR amplification of crtRB1 and lcyE genes were performed in the previously mentioned study, and based on the results, 10 inbred lines were used in crosses with 5 elite inbred lines (Table 8), in a factorial system. Following these crosses, 50 maize hybrids were generated.

Since the inbred lines identified as favorable for increasing the carotenoid content are older lines, they were used as paternal parents, while the elite lines, characterized by a good general combining ability, were used as maternal genotypes. The elite inbred lines are part of the group of lines currently used in the crossing programs at ARDS Turda due to their production capacity, tolerance to stress factors, and ability to transmit some favorable traits in hybrids. All 5 lines are parental forms of registered or perspective hybrids. It should also be mentioned that the lines were obtained from different initial materials, not being related to each other. Regarding paternal inbred lines, out of a total of 748 inbred lines analyzed in a previous study [2], 191 were homozygous for crtRB1, 8 for lcyE, and 4 had both genes. Lines from all three groups were sown, and crosses with the elite lines were carried out in the summer of 2021. A selection of genotypes was also made, based on their adaptive capacity, so that those that showed sensitivity to various stress factors (low temperatures, heat, pests, diseases) were not used in crosses. An attempt was made to achieve as many crosses as possible, but due to the lack of coincidence at anthesis between the two parental forms, some crosses could not be successfully achieved, or the seeds obtained were insufficient to carry out the experimentation. Also, in the case of some paternal lines, crosses were only possible with some of the maternal lines. This resulted in 50 hybrids that could be experimented with in a factorial system of the type 5 × 10.

### 4.2. Experimental Design

Both the inbred lines and the hybrids were tested in 2022 and 2023, in the experimental field of the Maize breeding laboratory of the ARDS Turda, located in the north-western part of Turda, Cluj County, Romania; the maize breeding field is situated on the upper terrace of the Aries River.

The hybrids were sown at the beginning of May, at a density of 70,000 plants/ha using the experimental sowing machine Monoseed DT (Wintersteiger, Ried im Innkreis, Austria). Each plot consisted of four rows of 5 m. The plots were arranged according to the randomized block design, with three replications The parental inbred lines were sown at a density of 60,000 plants/ha. To avoid cross-pollination, ten cobs from each inbred line and hybrid were covered before stigmata emergence, and were later manually self-pollinated; the resulting kernels were them mixed and milled, and carotenoid extraction was performed.

The experimental field followed a three-year crop rotation: soybean–winter wheat–maize. The field was plowed in the fall, and in the spring a pass was made with the combiner to level the land, when 400 kg ha^−1^ of N_20_:P_20_:K_0_ fertilizer was also applied. To control weeds, two herbicides were used: pre-emergence 1.5 L/ha a.s. S-metolachlor (960 g/L) and post-emergence 1.5 L/ha s.a. tembotrione (44 g/L) and isoxadiphen-ethyl (22 g/L).

### 4.3. Climatic Conditions

**Figure 3:**
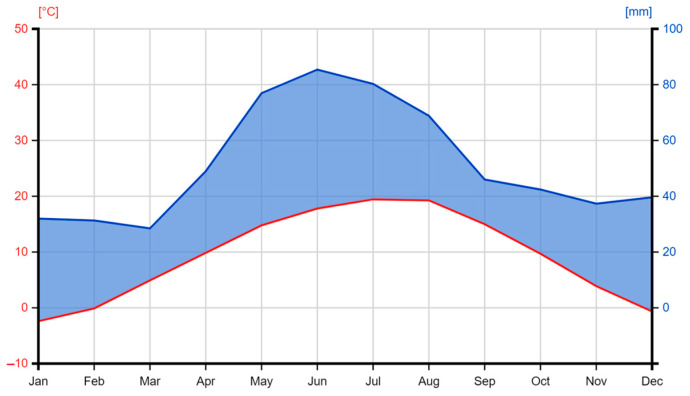
Specific climate of studied area, based on average temperatures (red line) and precipitation (blue line) from 1957–2022 [83].

Data on climatic conditions were obtained from the Turda Meteorological Station, located near the experimental fields. The climate in the Turda area is continental, with the highest temperatures in July and August and the heaviest precipitation occurring in June (Figure 3).

The year 2022 was one of the driest in recent decades. A water deficit associated with temperatures above the multi-year average was observed throughout the year (Figure S2). The sowing period was quite cool, but the precipitation levels were consistent with the typical conditions for the area, ensuring optimal conditions for crop emergence. Unfortunately, June and July were characterized by an extreme precipitation deficit, associated with high temperatures, causing a strong stress on plants’ optimal development [49]. Extreme temperatures in July, associated with the lack of precipitation, negatively impacted pollination and grain filling. Extreme temperatures were also recorded in August, and rainfall, although in large quantities, was recorded only in the last days of the month.

The year 2023 was similarly unfavorable for the maize crop due to adverse weather conditions: from the start of the year, temperatures exceeded the typical values for the area, with a particularly high thermal maximum recorded in January. Although June conditions were favorable for plant development, the high temperatures in July and August, combined with insufficient rainfall, negatively affected grain production. It should be noted that, in August, maximum temperatures of over 32 °C were recorded on 13 days, and precipitation was recorded especially in the last days of the month. Excessively high temperatures were also recorded in September and October, making these months unusually warm.

### 4.4. Grain Quality Determination

The analyses for the content of protein, fat, and fiber in the grain were performed using a Tango NIR spectrophotometer (Bruker, Billerica, MA, USA). For each genotype, 10 random cobs were chosen and ground, and a sample of approximately 100 g was analyzed. Each analysis was conducted in triplicate.

### 4.5. HPLC Determination of Carotenoids

Grain samples of ~50 g were milled using a WZ-1 mill (Sadkiewicz Instruments, Bydgoszcz—Poland). From the resulting flour, 1 g was used for carotenoid extraction. The extraction was performed using 50 mL of absolute ethanol, with the sample mixed for 120 min on an AM4 magnetic stirrer (Velp Scientifica, Usmate Velate, Italy); the resulting suspension was then filtered under vacuum through a G3 frit. An aliquot of the extract was evaporated under vacuum at 40 °C using a Laborota 4010 rotary evaporator (Heidolph Instruments, Schwabach, Germany). The residue was subsequently redissolved in 5 mL of acetone and filtered through a 0.47 µm membrane filter. The prepared sample was then subjected to high-performance liquid chromatography (HPLC) analysis, using a Flexar HPLC system (Perkin Elmer, Waltham, MA, USA) [3]. Three replicates of each sample were analyzed and the mean values were reported.

### 4.6. Data Processing and Statistical Methods

Data were analyzed using Past 4 for ANOVA. Fisher’s protected least significant difference (LSD) test was applied to evaluate the significance of the differences among the genotypes at *p*-values 0.05, 0.01, and 0.001.

GCA effects were calculated from the variances of paternal and maternal main effects, while SCA effects were inferred from the interactions between the two parental lines [84,85].

The additive effects (GCA) were calculated based on the formula:

$$\hat{g}m/\hat{g}p=X_{m/p}-\bar{X}$$

The non-additive effects (SCA) were calculated using the formula:

$${\hat{s}}_{mp}=X_{mp}-\bar{X}-\left(\hat{g}m+\hat{g}p\right)$$

where

X_m/p_ = mean of the m (maternal) or p (paternal) parent;
$\bar{X}$ = the mean of all values of the system;
X_mp_ = the value of mxp;
ĝm = GCA effects of the maternal inbred lines;
ĝp = GCA effects of the paternal lines;
ŝ_mp_ = SCA effects of the mxp crossings.

Mid-parent heterosis was calculated according to the formula [86]:

$$mpH=\frac{F1-mp}{mp}\times 100$$

where

mpH = the mid-parent heterosis, F1 = hybrid value, and mp = average value of the parents.

## 5. Conclusions

The paternal inbred line D302 proved a very good GCA for yield, surpassing the average with 1446 kg ha^−1^. The most productive hybrid in this study resulted from crossing this line with the elite maternal line E390, a hybrid that not only achieved the highest yield (9400 kg ha^−1^), but also exhibited a high content of zeaxanthin and cryptoxanthin; additionally, during the flowering period, this hybrid showed protogyny, even in unfavorable environmental conditions. Hybrids obtained by crossing the same paternal line with the maternal lines A452 (yield 8490 kg ha^−1^, total carotenoids 22.32 μg/g, lutein 9.75 μg/g and zeaxanthin 6.91 μg/g), A447 (yield 8866 kg ha^−1^, lutein 10.22 μg/g, unbroken plants 100%) and A483 (yield 8047 kg ha^−1^, β-carotene 0.90 μg/g, unbroken plants 97.7%) were also noted for their superior performance.

The hybrid A447×D302 (Figure S2) also stood out for its good production in both experimental years, also showing good tolerance to adverse conditions, especially through reduced ASI. This hybrid is proposed for further testing and for its possible registration for production in Romania.

## Figures and Tables

**Table 1:** Inbred lines carotenoid content.

Inbred Line	Total Carotenoids (μg/g)	Lutein (μg/g)	Zeaxanthin (μg/g)	Β-Cryptoxanthin (μg/g)	Β-Carotene (μg/g)
Maternal inbred lines					
E390	16.32	4.47	6.02	0.88	0.54
A447	18.92	8.38	7.07	1.55	0.52
A452	10.64	4.83	3.78	0.14	0.48
A483	19.08	5.33	6.85	1.42	0.65
E385	23.44	7.08	7.41	0.99	0.70
Paternal inbred lines					
T158	17.54	3.33	7.01	2.63	2.98
C382	21.76	5.00	7.83	4.79	3.05
D302	40.20	12.46	14.07	6.43	3.22
N4	29.82	19.68	2.68	2.68	2.98
LPN	2.30	0.46	0.69	0.35	0.35
LC28	30.46	8.23	11.58	4.57	2.13
LC54	7.52	2.89	3.25	1.44	0.72
LC51	13.38	4.28	4.55	1.74	1.34
LC62	32.12	4.18	15.74	4.18	1.28
Pi43-971	18.64	8.95	2.98	3.17	1.86

**Table 2:** GCA effects of studied maternal inbred lines.

Trait	Maternal Inbred Line									
	E390		A447		A452		A483		E385	
Yield	192	^ns^	125	^ns^	643	^ns^	470	^ns^	−1431	^0^
Erect plant	1.7	^ns^	1.3	^ns^	−4.8	^ns^	3.5	^ns^	−1.6	^ns^
Dry matter	−0.2	^ns^	−0.8	^ns^	0.3	^ns^	−1.4	^0^	2.0	*
Protein	−0.08	^ns^	−0.01	^ns^	−0.13	^ns^	0.05	^ns^	0.17	^ns^
Fat	−0.39	^ns^	0.02	^ns^	0.21	^ns^	0.70	*	−0.55	^ns^
Fiber	0.19	^ns^	0.10	^ns^	−0.01	^ns^	0.26	^ns^	−0.54	^0^
Total carotenoids	0.26	^ns^	−0.75	^ns^	0.08	^ns^	−0.06	^ns^	0.47	^ns^
Lutein	−0.54	^ns^	0.29	^ns^	1.01	*	−1.02	^0^	0.26	^ns^
Zeaxanthin	0.55	*	−1.05	^0^	−0.43	^ns^	0.23	^ns^	0.70	*
β-cryptoxanthin	0.15	^ns^	−0.10	^ns^	−0.21	^ns^	0.22	^ns^	−0.05	^ns^
β-carotene	0.10	^ns^	−0.11	^ns^	−0.14	^ns^	0.12	^ns^	0.04	^ns^

*/^0^ significant at *p* < 0.05, positive/negative values; ^ns^ not significant.

**Table 3:** GCA effects of studied paternal inbred lines.

Trait	Paternal Inbred Line																			
	T158		C382		D302		N4		LPN		LC28		LC51		LC57		LC62		Pi43-971	
Yield	−752	^ns^	−664	^ns^	1446	*	26	^ns^	33	^ns^	315	^ns^	−95	^ns^	117	^ns^	70	^ns^	−495	^ns^
Erect plant	−8.9	^0^	4.5	^ns^	1.8	^ns^	1.2	^ns^	2.2	^ns^	2.6	^ns^	0.6	^ns^	−5.9	^ns^	0.6	^ns^	1.6	^ns^
Dry matter	0.6	^ns^	1.0	^ns^	−0.6	^ns^	0.3	^ns^	−2.9	^0^	0.9	^ns^	−0.4	^ns^	0.03	^ns^	1.5	*	−0.4	^ns^
Protein	−0.06	^ns^	−0.48	^0^	−0.61	^0^	0.48	*	−0.30	^0^	0.60	*	−0.01	^ns^	0.47	*	0.38	*	−0.46	^0^
Fat	0.02	^ns^	−0.41	^ns^	−0.26	^ns^	0.17	^ns^	−0.12	^ns^	0.74	*	−0.20	^ns^	0.20	^ns^	−0.11	^ns^	−0.02	^ns^
Fiber	0.17	^ns^	−0.22	^ns^	−0.18	^ns^	−0.03	^ns^	0.33	^ns^	0.10	^ns^	−0.06	^ns^	−0.21	^ns^	0.06	^ns^	0.03	^ns^
Total carotenoids	−0.11	^ns^	1.13	^ns^	2.58	*	1.82	*	−7.37	^0^	1.46	^ns^	−1.00	^ns^	0.54	^ns^	2.34	*	−1.40	^ns^
Lutein	−0.64	^ns^	−0.16	^ns^	0.81	*	2.05	*	−3.30	^0^	0.30	^ns^	−0.51	^ns^	0.47	^ns^	−0.01	^ns^	0.98	*
Zeaxanthin	0.53	*	1.06	*	1.10	*	−0.44	^ns^	−2.55	^0^	0.52	*	−0.49	^ns^	0.03	^ns^	1.46	*	−1.21	^0^
β-cryptoxanthin	−0.08	^ns^	0.53	*	0.03	^ns^	0.03	^ns^	−0.16	^ns^	0.05	^ns^	−0.16	^ns^	−0.02	^ns^	0.18	^ns^	−0.39	^0^
β-carotene	0	^ns^	−0.04	^ns^	0.12	^ns^	0.11	^ns^	−0.17	^ns^	0.08	^ns^	−0.19	^ns^	0.08	^ns^	−0.02	^ns^	0.03	^ns^

*/^0^ significant at *p* < 0.05, positive/negative values; ^ns^ not significant.

**Table 4:** SCA of studied maize hybrids for yield and other agronomic traits.

Maternal Inbred Line	Trait	Paternal Inbred Line																			
		T158		C382		D302		N4		LPN		LC28		LC54		LC51		LC62		Pi43/971	
E390	ASI	1/1		0/1		−1/−2		0/2		4/3		1/2		1/1		0/0		2/1		2/0	
	Yield	6076	^000^	6864	^ns^	9400	***	7577	^ns^	6167	^00^	7457	^ns^	7801	*	7594	^ns^	7510	^ns^	6151	^00^
	UP	89.8	^ns^	98.6	^ns^	94.4	^ns^	97.3	^ns^	94.8	^ns^	96.0	^ns^	96.7	^ns^	89.0	^ns^	97.2	^ns^	96.0	^ns^
	DM	79.2	^ns^	79.0	^ns^	78.4	^ns^	78.4	^ns^	74.8	^000^	78.5	^ns^	76.7	^0^	77.3	^ns^	79.6	*	77.9	^ns^
A447	ASI	3/2		0/1		0/−1		0/0		1/1		2/0		1/1		2/1		−1/0		0/0	
	Yield	6773	^ns^	6954	^ns^	8866	***	6665	^ns^	7290	^ns^	8104	**	8135	**	7944	*	7239	^ns^	6312	^0^
	UP	84.4	^0^	100.0	^ns^	97.3	^ns^	96.1	^ns^	95.2	^ns^	95.8	^ns^	96.1	^ns^	90.4	^ns^	94.1	^ns^	95.8	^ns^
	DM	77.1	^ns^	78.8	^ns^	75.9	^000^	77.4	^ns^	75.4	^00^	79.0	^ns^	77.3	^ns^	77.8	^ns^	79.6	*	76.1	^00^
A452	ASI	2/1		−2/−2		0/−1		1/1		4/4		1/0		−1/−2		0/0		−2/−2		0/0	
	Yield	6625	^ns^	7447	^ns^	8490	***	7796	*	8511	***	7898	*	7581	^ns^	7809	*	7252	^ns^	7932	*
	UP	68.0	^000^	94.3	^ns^	93.3	^ns^	91.3	^ns^	91.9	^ns^	96.7	^ns^	85.7	^0^	82.7	^00^	88.5	^ns^	91.8	^ns^
	DM	79.3	^ns^	80.1	**	77.4	^ns^	78.1	^ns^	75.6	^000^	79.8	*	78.2	^ns^	78.9	^ns^	79.7	*	78.4	^ns^
A483	ASI	3/3		3/2		1/0		2/1		2/2		5/2		0/0		6/4		5/3		2/3	
	Yield	7150	^ns^	6524	^ns^	8047	*	7798	*	7472	^ns^	8119	**	8048	**	7076	^ns^	7949	*	7146	^ns^
	UP	92.6	^ns^	99.3	^ns^	97.7	^ns^	97.8	^ns^	98.7	^ns^	98.1	^ns^	97.2	^ns^	92.0	^ns^	95.8	^ns^	98.0	^ns^
	DM	77.0	^ns^	78.1	^ns^	77.6	^ns^	77.9	^ns^	73.1	^000^	76.9	^0^	75.8	^000^	76.6	^0^	78.7	^ns^	76.7	^0^
E385	ASI	1/1		1/1		2/1		3/2		7/4		1/1		0/0		0/0		1/1		2/2	
	Yield	4736	^000^	4924	^000^	7123	^ns^	6325	^0^	6253	^00^	5357	^000^	4986	^000^	5878	^000^	5954	^000^	4889	^000^
	UP	86.6	^ns^	96.4	^ns^	92.4	^ns^	89.6	^ns^	96.6	^ns^	92.5	^ns^	93.6	^ns^	82.6	^00^	93.8	^ns^	92.9	^ns^
	DM	81.4	***	80.1	**	78.9	^ns^	80.5	***	77.6	^ns^	81.1	***	81.2	***	80.6	***	80.8	***	80.1	**

*,**,***/^0^,^00^,^000^ significant at *p* < 0.05, *p* < 0.01, and *p* < 0.001; ^ns^ not significant; ASI = anthesis to silking interval, UP = unbroken plants, DM = dry matter.

**Table 5:** SCA of studied inbred lines for protein, fat, and fiber.

Maternal Inbred Line	Trait	Paternal Inbred Line																			
		T158		C382		D302		N4		LPN		LC28		LC54		LC51		LC62		Pi43/971	
E390	Protein	8.28	^000^	7.58	^000^	8.32	^000^	9.30	**	8.41	^00^	9.41	***	8.49	^0^	9.74	***	9.39	***	8.84	^ns^
	Fat	2.87	^ns^	2.55	^00^	2.65	^0^	3.00	^ns^	2.77	^ns^	3.56	^ns^	2.71	^0^	3.26	^ns^	2.84	^ns^	3.15	^ns^
	Fiber	2.76	***	2.46	^ns^	2.51	^ns^	2.42	^ns^	2.45	^ns^	2.65	^ns^	2.55	^ns^	2.29	^ns^	2.93	**	2.41	^ns^
A447	Protein	9.55	***	8.64	^ns^	8.30	^000^	9.56	***	8.72	^ns^	8.73	^ns^	9.49	***	9.22	*	8.27	^000^	8.02	^000^
	Fat	3.68	^ns^	3.14	^ns^	2.67	^0^	3.08	^ns^	3.23	^ns^	4.25	**	3.21	^ns^	3.78	^ns^	3.45	^ns^	3.00	^ns^
	Fiber	2.55	^ns^	2.39	^ns^	2.14	^ns^	2.03	^ns^	2.72	*	2.65	^ns^	2.29	^ns^	2.40	^ns^	2.92	**	2.46	^ns^
A452	Protein	7.93	^000^	7.78	^000^	8.14	^000^	8.91	^ns^	9.41	***	9.45	***	8.44	^00^	9.18	*	9.57	***	8.40	^00^
	Fat	3.50	^ns^	3.26	^ns^	3.23	^ns^	3.85	^ns^	3.38	^ns^	4.14	**	2.77	^ns^	3.85	^ns^	3.92	*	3.47	^ns^
	Fiber	2.59	^ns^	2.30	^ns^	1.96	^0^	2.33	^ns^	2.55	^ns^	2.38	^ns^	2.18	^ns^	2.18	^ns^	2.58	^ns^	2.33	^ns^
A483	Protein	9.63	***	8.22	^000^	8.60	^ns^	9.39	***	8.23	^000^	9.16	*	8.82	^ns^	9.12	^ns^	9.68	***	8.22	^000^
	Fat	4.30	**	3.23	^ns^	3.85	^ns^	4.05	*	4.20	**	4.93	***	4.45	***	4.25	**	3.54	^ns^	3.51	^ns^
	Fiber	2.94	^ns^	2.42	^ns^	2.30	^ns^	2.58	^ns^	3.27	^ns^	2.82	^ns^	2.42	^ns^	2.73	*	2.24	^ns^	2.40	^ns^
E385	Protein	8.59	^ns^	9.66	***	7.88	^000^	9.52	***	7.99	^000^	10.52	***	8.95	^ns^	9.39	***	9.26	**	8.51	^0^
	Fat	2.36	^00^	2.40	^00^	2.92	^ns^	3.52	^ns^	2.45	^00^	3.45	^ns^	2.47	^00^	2.48	^00^	2.33	^00^	3.42	^ns^
	Fiber	1.77	^00^	1.07	^000^	1.94	^0^	2.25	^ns^	2.44	^ns^	1.75	^00^	2.03	^ns^	1.12	^000^	1.39	^000^	2.32	^ns^

*,**,***/^0^,^00^,^000^ significant at *p* < 0.05, *p* < 0.01, and *p* < 0.001; ^ns^ not significant.

**Table 6:** SCA of studied inbred lines for carotenoid content.

Maternal Inbred Line	Trait	Paternal Inbred Line																			
		T158		C382		D302		N4		LPN		LC28		LC54		LC51		LC62		Pi43/971	
E390	TC	15.02	^00^	17.33	^ns^	17.82	^ns^	20.08	**	7.51	^000^	19.86	**	16.64	^ns^	20.20	***	25.08	***	16.03	^ns^
	Lut	5.13	^000^	5.60	^00^	5.46	^00^	7.57	*	1.95	^000^	7.75	**	4.94	^000^	6.12	^ns^	8.62	***	7.87	**
	Zeax	6.40	***	6.73	***	6.98	***	5.78	^ns^	2.58	^000^	6.45	***	4.71	^000^	7.27	***	9.34	***	5.07	^0^
	β-crypt	0.95	^ns^	1.61	***	1.39	*	1.45	**	0.78	^ns^	1.23	^ns^	0.63	^0^	1.29	^ns^	1.50	**	0.67	^0^
	β-carot	0.78	^ns^	0.46	^ns^	0.71	^ns^	1.04	***	0.41	^ns^	0.82	*	0.43	^ns^	1.24	***	0.67	^ns^	0.42	^ns^
A447	TC	15.81	^ns^	18.68	^ns^	21.85	***	18.56	^ns^	7.45	^000^	18.71	^ns^	14.88	^00^	16.84	^ns^	16.53	^ns^	16.13	^ns^
	Lut	6.10	^ns^	7.20	^ns^	10.22	***	10.03	***	2.88	^000^	6.20	^ns^	5.33	^000^	6.95	^ns^	5.79	^0^	8.60	***
	Zeax	4.57	^000^	5.52	^ns^	6.45	***	3.58	^000^	2.02	^000^	5.10	^ns^	4.86	^00^	4.05	^000^	5.63	^ns^	3.49	^000^
	β-crypt	0.80	^ns^	1.29	^ns^	1.23	^ns^	0.67	^0^	0.43	^000^	0.93	^ns^	0.97	^ns^	0.92	^ns^	1.16	^ns^	0.61	^0^
	β-carot	0.41	^ns^	0.48	^ns^	0.56	^ns^	0.27	^00^	0.25	^00^	0.89	*	0.38	^0^	0.52	^ns^	0.41	^ns^	0.68	^ns^
A452	TC	16.01	^ns^	19.11	*	22.32	***	20.75	***	7.48	^000^	19.96	**	14.51	^00^	17.54	^ns^	18.65	^ns^	17.39	^ns^
	Lut	5.77	^0^	7.77	**	9.75	***	11.56	***	2.00	^000^	9.11	***	7.48	*	7.84	**	6.86	^ns^	8.40	**
	Zeax	5.59	^ns^	7.22	***	6.91	***	4.69	^000^	1.71	^000^	6.62	***	4.43	^000^	4.44	^000^	5.70	^ns^	4.23	^000^
	β-crypt	0.21	^000^	1.51	**	0.95	^ns^	0.70	^ns^	0.38	^000^	1.22	^ns^	0.61	^0^	0.68	^0^	0.85	^ns^	0.80	^ns^
	β-carot	0.41	^ns^	0.48	^ns^	0.53	^ns^	0.57	^ns^	0.22	^00^	0.50	^ns^	0.37	^0^	0.48	^ns^	0.53	^ns^	0.46	^ns^
A483	TC	21.51	***	20.06	**	20.30	***	17.17	^ns^	14.55	^00^	15.19	^0^	14.78	^00^	15.35	^0^	18.04	^ns^	15.42	^0^
	Lut	7.06	^ns^	5.98	^ns^	6.03	^ns^	6.04	^ns^	4.99	^000^	4.05	^000^	4.71	^000^	5.49	^00^	4.97	^000^	6.87	^ns^
	Zeax	7.55	***	7.23	***	5.91	^ns^	6.37	**	5.03	^0^	5.09	^ns^	4.61	^000^	4.69	^000^	6.78	***	4.88	^00^
	β-crypt	1.59	***	2.10	***	0.62	^0^	1.48	**	1.48	**	0.94	^ns^	1.08	^ns^	1.03	^ns^	1.42	*	0.45	^000^
	β-carot	0.94	**	0.92	**	0.90	**	0.67	^ns^	0.53	^ns^	0.69	^ns^	0.34	^0^	0.65	^ns^	0.78	^ns^	0.76	^ns^
E385	TC	17.56	^ns^	16.95	^ns^	17.09	^ns^	19.01	*	12.64	^000^	20.07	**	20.65	***	19.22	*	19.89	**	14.52	^00^
	Lut	5.95	^ns^	5.87	^0^	5.78	^0^	8.28	***	4.90	^000^	7.58	*	8.19	***	9.15	***	6.94	^ns^	6.39	^ns^
	Zeax	6.43	**	6.50	***	7.18	***	5.29	^ns^	3.82	^000^	7.23	***	6.84	***	7.62	***	7.75	***	4.17	^000^
	β-crypt	1.07	^ns^	1.14	^ns^	0.95	^ns^	0.87	^ns^	1.13	^ns^	0.93	^ns^	0.91	^ns^	0.97	^ns^	1.00	^ns^	0.53	^00^
	β-carot	0.46	^ns^	0.44	^ns^	0.89	*	0.97	**	0.74	^ns^	0.51	^ns^	0.54	^ns^	0.49	^ns^	0.52	^ns^	0.83	*

*/^0^ significant at *p* < 0.05, positive/negative values; **/^00^ significant at *p* < 0.01, positive/negative values; ***/^000^ significant at *p* < 0.001, positive/negative; ^ns^ not significant; TC = total carotenoids, Lut = lutein, Zeax = zeaxanthin, β-crypt = β-cryptoxanthin, β-carot = β-carotene.

**Table 7:** MPH% for carotenoid content.

Maternal Inbred Line	Paternal Inbred Line										
	Trait	T158	C382	D302	N4	LPN	LC28	LC54	LC51	LC62	Pi43/971
E390	TC	−11.3	−9.0	−36.9	−13.0	−19.3	−15.1	39.6	36.0	3.6	−8.3
	Lut	31.5	18.3	−35.5	−37.3	−20.9	22.0	34.2	39.9	99.3	17.3
	Zeax	−1.8	−2.8	−30.5	32.9	−23.1	−26.7	1.6	37.6	−14.2	12.7
	β-crypt	−45.9	−43.2	−62.0	−18.5	26.8	−54.9	−45.7	−1.5	−40.7	−66.9
	β-carot	−55.7	−74.4	−62.2	−40.9	−7.9	−38.6	−31.7	31.9	−26.4	−65.0
A447	TC	−13.3	−8.2	−26.1	−23.8	−29.8	−24.2	12.6	4.3	−35.2	−14.1
	Lut	4.2	7.6	−1.9	−28.5	−34.8	−25.3	−5.4	9.8	−7.8	−0.8
	Zeax	−35.1	−25.9	−39.0	−26.6	−47.9	−45.3	−5.8	−30.3	−50.6	−30.5
	β-crypt	−61.7	−59.3	−69.2	−68.3	−54.7	−69.6	−35.1	−44.1	−59.5	−74.2
	β-carot	−76.6	−73.1	−70.1	−84.6	−42.5	−32.8	−38.7	−44.1	−54.4	−42.9
A452	TC	13.6	18.0	−12.2	2.6	15.6	−2.9	59.8	46.0	−12.8	18.8
	Lut	41.4	58.1	12.8	−5.7	−24.4	39.5	93.8	72.1	52.3	21.9
	Zeax	3.6	24.4	−22.6	45.2	−23.5	−13.8	26.0	6.6	−41.6	25.1
	β-crypt	−84.8	−38.7	−71.1	−50.4	55.1	−48.2	−22.8	−27.7	−60.6	−51.7
	β-carot	−76.3	−72.8	−71.4	−67.1	−47.0	−61.7	−38.3	−47.3	−39.8	−60.7
A483	TC	17.5	−1.8	−31.5	−29.8	36.1	−38.7	11.1	−5.4	−29.5	−18.2
	Lut	63.0	15.8	−32.2	−51.7	72.4	−40.3	14.6	14.3	4.5	−3.8
	Zeax	8.9	−1.5	−43.5	33.7	33.4	−44.8	−8.7	−17.7	−40.0	−0.7
	β-crypt	−21.5	−32.4	−84.2	−27.8	67.2	−68.6	−24.5	−34.8	−49.3	−80.4
	β-carot	−48.2	−50.3	−53.5	−63.1	6.0	−50.4	−50.4	−34.7	−19.2	−39.4
E385	TC	−14.3	−25.0	−46.3	−28.6	−1.8	−25.5	33.4	4.4	−28.4	−31.0
	Lut	14.3	−2.8	−40.8	−38.1	30.0	−1.0	64.3	61.1	23.3	−20.3
	Zeax	−10.8	−14.7	−33.1	4.9	−5.7	−23.9	28.3	27.4	−33.0	−19.7
	β-crypt	−40.9	−60.6	−74.4	−52.6	68.7	−66.5	−25.1	−28.9	−61.3	−74.5
	β-carot	−75.0	−76.5	−54.6	−47.3	41.0	−64.0	−23.9	−52.0	−47.5	−35.2

TC = total carotenoids, Lut = lutein, Zeax = zeaxanthin, β-crypt = β-cryptoxanthin, β-carot = β-carotene.

**Table 8:** Inbred lines used in crosses and their cob traits.

Inbred Line	Homozygous for	Kernel Type	Kernel Color
Maternal inbred lines			
E390	-	Flint	Dark yellow
A447	-	Dent	Dark yellow
A452	-	Dent ×flint	Dark yellow
A483	-	Dent	Dark yellow
E385	-	Dent	Yellow
Paternal inbred lines			
T158	crtRB1 + lcyE	Dent × flint	Dark yellow
C382	lcyE	Dent × flint	Yellow
D302	crtRB1	Dent	Dark yellow
N4	crtRB1	Dent × flint	Light yellow
LPN	lcyE	Dent × flint	White and yellow
LC28	lcyE	Dent × flint	Dark yellow
LC54	lcyE	Dent × flint	Yellow
LC51	lcyE	Dent × flint	Yellow
LC62	lcyE	Dent	Yellow
Pi43-971	crtRB1	Dent	Light yellow

## Data Availability

The original contributions presented in this study are included in the article/Supplementary Material. Further inquiries can be directed to the corresponding author.

## Supplementary Materials

The following supporting information can be downloaded at: https://www.mdpi.com/article/10.3390/plants14010138/s1, Figure S1: Ears and plants of A447×D302 hybrid; Figure S2: Temperature and precipitation deviation compared to 65-year average (Turda, 2022–2023).

## References

[1] (2024). FAOSTAT. https://www.fao.org/faostat/en/#data/QCL.

[2] Băcilă I., Haș V., Șuteu D., Miclăuș M., Coste A., Muntean E., Vana C.D., Varga A., Călugăr R., Copândean A. (2022). Screening of the Romanian Maize (*Zea mays* L.) Germplasm for crtRB1 and lcyE Alleles Enhancing the Provitamin A Concentration in Endosperm. Not. Bot. Horti Agrobot. Cluj-Napoca.

[3] Calugar R.E., Muntean E., Varga A., Vana C.D., Has V.V., Tritean N., Ceclan L.A. (2022). Improving the Carotenoid Content in Maize by Using Isonuclear Lines. Plants.

[4] Scrob S., Muste S., Haș I., Mureșan C., Socaci S., Fărcaș A. (2014). Total Content of Carotenoids in Corn Landraces and Their Potential Health Applications. Bull. Univ. Agric. Sci. Veter- Med. Cluj-Napoca. Food Sci. Technol..

[5] Muntean E. Carotenoids in Several Transylvanian Maize Hybrids. Proceedings of the 1st International Electronic Conference on Plant Science.

[6] Gao H., Gadlage M.J., Lafitte H.R., Lenderts B., Yang M., Schroder M., Farrell J., Snopek K., Peterson D., Feigenbutz L. (2020). Superior Field Performance of Waxy Corn Engineered Using CRISPR–Cas9. Nat. Biotechnol..

[7] Obadi M., Qi Y., Xu B. (2023). High-Amylose Maize Starch: Structure, Properties, Modifications and Industrial Applications. Carbohydr. Polym..

[8] Wu Y., Campbell M., Yen Y., Wicks Z., Ibrahim A.M.H. (2009). Genetic Analysis of High Amylose Content in Maize (*Zea mays* L.) Using a Triploid Endosperm Model. Euphytica.

[9] Subaedah S., Edy E., Mariana K. (2021). Growth, Yield, and Sugar Content of Different Varieties of Sweet Corn and Harvest Time. Int. J. Agron..

[10] Revilla P., Anibas C.M., Tracy W.F. (2021). Sweet Corn Research around the World 2015–2020. Agronomy.

[11] Harakotr B., Sutthiluk W., Rithichai P. (2022). Changes on Sugar and Starch Contents during Seed Development of Synergistic Sweet Corn and Implication on Seed Quality. Int. J. Agron..

[12] Inplean C., Jompuk P., Chai-Arree W., Stamp P., Jompuk C. (2020). Improved Sugar Content in a Sweet Corn Grain Mutant with High Quality Protein and Anthocyanin. Agric. Nat. Resour..

[13] Djalovic I., Grahovac N., Stojanović Z., Đurović A., Živančev D., Jakšić S., Jaćimović S., Tian C., Prasad P.V.V. (2024). Nutritional and Chemical Quality of Maize Hybrids from Different FAO Maturity Groups Developed and Grown in Serbia. Plants.

[14] Shawa H., Van Biljon A., Labuschagne M.T. (2021). Protein Quality and Quantity of Quality Protein Maize (QPM) and non-QPM Hybrids under Optimal and Low Nitrogen Conditions. Cereal Chem..

[15] Amegbor I.K., van Biljon A., Shargie N., Tarekegne A., Labuschagne M.T. (2022). Heritability and Associations among Grain Yield and Quality Traits in Quality Protein Maize (QPM) and Non-QPM Hybrids. Plants.

[16] Maqbool M.A., Beshir Issa A., Khokhar E.S. (2021). Quality Protein Maize (QPM): Importance, Genetics, Timeline of Different Events, Breeding Strategies and Varietal Adoption. Plant Breed..

[17] Ochieng’ I.O., Gitari H.I., Mochoge B., Rezaei-Chiyaneh E., Gweyi-Onyango J.P. (2021). Optimizing Maize Yield, Nitrogen Efficacy and Grain Protein Content under Different N Forms and Rates. J. Soil. Sci. Plant Nutr..

[18] Chandrasekharan N., Ramanathan N., Pukalenthy B., Chandran S., Manickam D., Adhimoolam K., Nalliappan G.K., Manickam S., Rajasekaran R., Sampathrajan V. (2022). Development of β-Carotene, Lysine, and Tryptophan-Rich Maize (*Zea mays*) Inbreds through Marker-Assisted Gene Pyramiding. Sci. Rep..

[19] Talukder Z.A., Muthusamy V., Chhabra R., Gain N., Reddappa S.B., Mishra S.J., Kasana R., Bhatt V., Chand G., Katral A. (2022). Combining Higher Accumulation of Amylopectin, Lysine and Tryptophan in Maize Hybrids through Genomics-Assisted Stacking of Waxy1 and Opaque2 Genes. Sci. Rep..

[20] Shetti P., Sagare D.B., Surender M., Reddy S.S. (2020). Development of Lysine and Tryptophan Rich Maize (*Zea mays*) Inbreds Employing Marker Assisted Backcross Breeding. Plant Gene.

[21] Das A.K., Gowda M.M., Muthusamy V., Zunjare R.U., Chauhan H.S., Baveja A., Bhatt V., Chand G., Bhat J.S., Guleria S.K. (2021). Development of Maize Hybrids With Enhanced Vitamin-E, Vitamin-A, Lysine, and Tryptophan Through Molecular Breeding. Front. Plant Sci..

[22] Huang S., Adams W.R., Zhou Q., Malloy K.P., Voyles D.A., Anthony J., Kriz A.L., Luethy M.H. (2004). Improving Nutritional Quality of Maize Proteins by Expressing Sense and Antisense Zein Genes. J. Agric. Food Chem..

[23] Du Q., Li W. (2024). Iron Biofortification in Maize by *ZmNAC78* Is a Promising and Sustainable Way to Fight Iron-deficiency Anaemia. Clin. Transl. Med..

[24] Ashraf M.A., Bakirbas A. (2023). Ironing out Biofortification in Maize: NAC78 Regulates Iron Concentrations in Kernels. Mol. Plant.

[25] Pillay K., Siwela M., Derera J., Veldman F.J. (2014). Provitamin A Carotenoids in Biofortified Maize and Their Retention during Processing and Preparation of South African Maize Foods. J. Food Sci. Technol..

[26] Dube N., Mashurabad P.C., Hossain F., Pullakhandam R., Thingnganing L., Bharatraj D.K. (2018). β-Carotene Bioaccessibility from Biofortified Maize (*Zea mays*) Is Related to Its Density and Is Negatively Influenced by Lutein and Zeaxanthin. Food Funct..

[27] Bouis H.E., Welch R.M. (2010). Biofortification—A Sustainable Agricultural Strategy for Reducing Micronutrient Malnutrition in the Global South. Crop Sci..

[28] Gupta H.S., Hossain F., Muthusamy V. (2015). Biofortification of Maize: An Indian Perspective. Indian J. Genet. Plant Breed..

[29] Ortiz-Monasterio J.I., Palacios-Rojas N., Meng E., Pixley K., Trethowan R., Peña R.J. (2007). Enhancing the Mineral and Vitamin Content of Wheat and Maize through Plant Breeding. J. Cereal Sci..

[30] Bouis H., Biesalski H.K., Birner R. (2018). Reducing Mineral and Vitamin Deficiencies through Biofortification: Progress Under HarvestPlus. World Review of Nutrition and Dietetics.

[31] Ahmad A., Riaz S., Shahzaib Nadeem M., Mubeen U., Maham K., María Martínez-Espinosa R. (2022). Role of Carotenoids in Cardiovascular Disease. Physiology.

[32] Leermakers E.T., Darweesh S.K., Baena C.P., Moreira E.M., Melo Van Lent D., Tielemans M.J., Muka T., Vitezova A., Chowdhury R., Bramer W.M. (2016). The Effects of Lutein on Cardiometabolic Health across the Life Course: A Systematic Review and Meta-Analysis. Am. J. Clin. Nutr..

[33] Maria A.G., Graziano R., Nicolantonio D. (2015). Carotenoids: Potential Allies of Cardiovascular Health?. Food Nutr. Res..

[34] García-Castro A., Román-Gutiérrez A.D., Castañeda-Ovando A., Cariño-Cortés R., Acevedo-Sandoval O.A., López-Perea P., Guzmán-Ortiz F.A. (2022). Cereals as a Source of Bioactive Compounds with Anti-Hypertensive Activity and Their Intake in Times of COVID-19. Foods.

[35] Shafe M.O., Gumede N.M., Nyakudya T.T., Chivandi E. (2024). Lycopene: A Potent Antioxidant with Multiple Health Benefits. J. Nutr. Metab..

[36] Rowles J.L., Erdman J.W. (2020). Carotenoids and Their Role in Cancer Prevention. Biochim. Et Biophys. Acta (BBA)—Mol. Cell Biol. Lipids.

[37] Gong X., Smith J., Swanson H., Rubin L. (2018). Carotenoid Lutein Selectively Inhibits Breast Cancer Cell Growth and Potentiates the Effect of Chemotherapeutic Agents through ROS-Mediated Mechanisms. Molecules.

[38] Smorowska A.J., Żołnierczyk A.K., Nawirska-Olszańska A., Sowiński J., Szumny A. (2021). Nutritional Properties and In Vitro Antidiabetic Activities of Blue and Yellow Corn Extracts: A Comparative Study. J. Food Qual..

[39] Chew B.P. (1993). Role of Carotenoids in the Immune Response. J. Dairy Sci..

[40] Roe D.A., Fuller C.J., Klurfeld D.M. (1993). Carotenoids and Immune Function. Nutrition and Immunology.

[41] Abdel-Aal E.-S., Akhtar H., Zaheer K., Ali R. (2013). Dietary Sources of Lutein and Zeaxanthin Carotenoids and Their Role in Eye Health. Nutrients.

[42] Bone R.A., Landrum J.T., Friedes L.M., Gomez C.M., Kilburn M.D., Menendez E., Vidal I., Wang W. (1997). Distribution of Lutein and Zeaxanthin Stereoisomers in the Human Retina. Exp. Eye Res..

[43] Johra F.T., Bepari A.K., Bristy A.T., Reza H.M. (2020). A Mechanistic Review of β-Carotene, Lutein, and Zeaxanthin in Eye Health and Disease. Antioxidants.

[44] Stahl W., Sies H. (2003). Antioxidant Activity of Carotenoids. Mol. Asp. Med..

[45] Dewanjee S., Bhattacharjee N., Chakraborty P., Bhattacharjee S., Zia-Ul-Haq M., Dewanjee S., Riaz M. (2021). Carotenoids as Antioxidants. Carotenoids: Structure and Function in the Human Body.

[46] Harjes C.E., Rocheford T.R., Bai L., Brutnell T.P., Kandianis C.B., Sowinski S.G., Stapleton A.E., Vallabhaneni R., Williams M., Wurtzel E.T. (2008). Natural Genetic Variation in Lycopene Epsilon Cyclase Tapped for Maize Biofortification. Science.

[47] Menkir A., Liu W., White W.S., Maziya-Dixon B., Rocheford T. (2008). Carotenoid Diversity in Tropical-Adapted Yellow Maize Inbred Lines. Food Chem..

[48] Da Silva Messias R., Galli V., Dos Anjos e Silva S.D., Rombaldi C.V. (2014). Carotenoid Biosynthetic and Catabolic Pathways: Gene Expression and Carotenoid Content in Grains of Maize Landraces. Nutrients.

[49] Călugăr R.E., Varga A., Vana C.D., Ceclan L.A., Racz I., Chețan F., Șimon A., Popa C., Tritean N., Russu F. (2024). Influence of Changing Weather on Old and New Maize Hybrids: A Case Study in Romania. Plants.

[50] Iordan H.L., Horhocea D., Martura T., Ciocăzanu I., Băduț C. (2022). Creation of maize hybrids for early sowing, with superior adaptability to adverse climatic conditions, with high agronomic performances. Analele Institutului Natl. Cercet.-Dezvoltare Agric. Fundulea.

[51] Horhocea D., Martura T., Iordan H.L., Băduț C., Ciocăzanu I., Lazăr C. (2022). Genetic progress in maize for drought resistance by shortening the vegetation period. Analele Institutului Natl. Cercet.-Dezvoltare Agric. Fundulea.

[52] Horhocea D., Martura T., Iordan H.L., Băduț C., Ciocăzanu I. (2021). Magnus, semi-early maize hybrid, released by the NARDI Fundulea. Analele Institutului Natl. Cercet.-Dezvoltare Agric. Fundulea.

[53] Panda A.O., Buzna C.C., Bulai A. (2020). The Evolution of Seed Production in Dioecious Hemp in The Period 2016-2018, In Climate Conditions from S.C.D.A. Lovrin. Life Sci. Sustain. Dev..

[54] Ruja A., Toma I., Bulai A., Agapie A.L., Negruț G., Suhai K., Gorinoiu G. (2021). The Impact of Climate Changes on Production in The Autumn and Spring Oats. Life Sci. Sustain. Dev..

[55] Ruja A., Gorinoiu G., Suhai K.R., Agapie A.L., Sala F., Schiller C.-M.I. (2022). The Effect of Climate Conditions on The Phenological Features of The Autumn Oat Crop. Life Sci. Sustain. Dev..

[56] Hălbac-Cotoară-Zamfir R., Salvati L., Eslamian S. (2023). Irrigation Management in Romania. Handbook of Irrigation Hydrology and Management.

[57] Rao A., Rao L. (2007). Carotenoids and Human Health. Pharmacol. Res..

[58] Maoka T. (2020). Carotenoids as Natural Functional Pigments. J. Nat. Med..

[59] Gebregziabher B.S., Gebremeskel H., Debesa B., Ayalneh D., Mitiku T., Wendwessen T., Habtemariam E., Nur S., Getachew T. (2023). Carotenoids: Dietary Sources, Health Functions, Biofortification, Marketing Trend and Affecting Factors—A Review. J. Agric. Food Res..

[60] Toti E., Chen C.-Y.O., Palmery M., Villaño Valencia D., Peluso I. (2018). Non-Provitamin A and Provitamin A Carotenoids as Immunomodulators: Recommended Dietary Allowance, Therapeutic Index, or Personalized Nutrition?. Oxidative Med. Cell. Longev..

[61] Zurak D., Grbeša D., Duvnjak M., Kiš G., Međimurec T., Kljak K. (2021). Carotenoid Content and Bioaccessibility in Commercial Maize Hybrids. Agriculture.

[62] Kuhnen S., Menel Lemos P.M., Campestrini L.H., Ogliari J.B., Dias P.F., Maraschin M. (2011). Carotenoid and Anthocyanin Contents of Grains of Brazilian Maize Landraces: Carotenoids and Anthocyanins of Brazilian Maize Landraces. J. Sci. Food Agric..

[63] Hwang T., Ndolo V.U., Katundu M., Nyirenda B., Bezner-Kerr R., Arntfield S., Beta T. (2016). Provitamin A Potential of Landrace Orange Maize Variety (*Zea mays* L.) Grown in Different Geographical Locations of Central Malawi. Food Chem..

[64] Wilson Reichert Júnior F., Rafael Silva De Oliveira C., Henrique Da Silva Júnior A., Mulinari J. (2020). Chemical Composition of Maize Landraces and Their Importance to Human Health. Ciência, Tecnologia e Inovação: Do campo à mesa.

[65] Eggersdorfer M., Wyss A. (2018). Carotenoids in Human Nutrition and Health. Arch. Biochem. Biophys..

[66] Zuma M., Kolanisi U., Modi A. (2018). The Potential of Integrating Provitamin A-Biofortified Maize in Smallholder Farming Systems to Reduce Malnourishment in South Africa. Int. J. Environ. Res. Public Health.

[67] Burt A.J., Grainger C.M., Young J.C., Shelp B.J., Lee E.A. (2010). Impact of Postharvest Handling on Carotenoid Concentration and Composition in High-Carotenoid Maize (*Zea mays* L.) Kernels. J. Agric. Food Chem..

[68] Duo H., Hossain F., Muthusamy V., Zunjare R.U., Goswami R., Chand G., Mishra S.J., Chhabra R., Gowda M.M., Pal S. (2021). Development of Sub-Tropically Adapted Diverse Provitamin-A Rich Maize Inbreds through Marker-Assisted Pedigree Selection, Their Characterization and Utilization in Hybrid Breeding. PLoS ONE.

[69] Liu L., Jeffers D., Zhang Y., Ding M., Chen W., Kang M.S., Fan X. (2015). Introgression of the crtRB1 Gene into Quality Protein Maize Inbred Lines Using Molecular Markers. Mol. Breed..

[70] Zunjare R.U., Hossain F., Muthusamy V., Baveja A., Chauhan H.S., Bhat J.S., Thirunavukkarasu N., Saha S., Gupta H.S. (2018). Development of Biofortified Maize Hybrids through Marker-Assisted Stacking of β-Carotene Hydroxylase, Lycopene-ε-Cyclase and Opaque2 Genes. Front. Plant Sci..

[71] Menkir A., Maziya-Dixon B., Mengesha W., Rocheford T., Alamu E.O. (2017). Accruing Genetic Gain in Pro-Vitamin A Enrichment from Harnessing Diverse Maize Germplasm. Euphytica.

[72] Zunjare R.U., Chhabra R., Hossain F., Muthusamy V., Baveja A., Gupta H.S. (2018). Development and Validation of Multiplex-PCR Assay for Simultaneous Detection of Rare Alleles of crtRB1 and lcyE Governing Higher Accumulation of Provitamin A in Maize. J. Plant Biochem. Biotechnol..

[73] Babu R., Rojas N.P., Gao S., Yan J., Pixley K. (2013). Validation of the Effects of Molecular Marker Polymorphisms in LcyE and CrtRB1 on Provitamin A Concentrations for 26 Tropical Maize Populations. Theor. Appl. Genet..

[74] Azmach G., Gedil M., Menkir A., Spillane C. (2013). Marker-Trait Association Analysis of Functional Gene Markers for Provitamin A Levels across Diverse Tropical Yellow Maize Inbred Lines. BMC Plant Biol..

[75] Gebremeskel S., Garcia-Oliveira A.L., Menkir A., Adetimirin V., Gedil M. (2018). Effectiveness of Predictive Markers for Marker Assisted Selection of Pro-Vitamin A Carotenoids in Medium-Late Maturing Maize (*Zea mays* L.) Inbred Lines. J. Cereal Sci..

[76] Muthusamy V., Hossain F., Thirunavukkarasu N., Choudhary M., Saha S., Bhat J.S., Prasanna B.M., Gupta H.S. (2014). Development of β-Carotene Rich Maize Hybrids through Marker-Assisted Introgression of β-Carotene Hydroxylase Allele. PLoS ONE.

[77] Sarca T., Cosmin O., Antohe I. (2007). Cercetări Şi Realizări În Ameliorarea Porumbului La Fundulea. Analele Institutului Natl. Cercet.-Dezvoltare Agric. Fundulea.

[78] Suwarno W.B., Pixley K.V., Palacios-Rojas N., Kaeppler S.M., Babu R. (2014). Formation of Heterotic Groups and Understanding Genetic Effects in a Provitamin A Biofortified Maize Breeding Program. Crop Sci..

[79] Barker T., Campos H., Cooper M., Dolan D., Edmeades G., Habben J., Schussler J., Wright D., Zinselmeier C. (2005). Improving Drought Tolerance in Maize. Plant Breeding Reviews.

[80] Fahad S., Bajwa A.A., Nazir U., Anjum S.A., Farooq A., Zohaib A., Sadia S., Nasim W., Adkins S., Saud S. (2017). Crop Production under Drought and Heat Stress: Plant Responses and Management Options. Front. Plant Sci..

[81] Wang L., Yan Y., Lu W., Lu D. (2021). Application of Exogenous Phytohormones at Silking Stage Improve Grain Quality under Post-Silking Drought Stress in Waxy Maize. Plants.

[82] Kim K.-H., Lee B.-M. (2023). Effects of Climate Change and Drought Tolerance on Maize Growth. Plants.

[83] Zepner L., Karrasch P., Wiemann F., Bernard L. (2021). ClimateCharts.Net—An Interactive Climate Analysis Web Platform. Int. J. Digit. Earth.

[84] Căbulea I. (2004). Genetica Porumbului. Porumbul—Studiu Monografic.

[85] Varga A., Călugăr R.E., Vana C., Ceclan L., Racz I., Tritean N. (2023). Assessment of the Degree of Relatedness of Some Inbred Lines Created at ARDS Turda. Agronomy.

[86] Hallauer A.R., Carena M.J., Filho J.B.M., Carena M.J., Hallauer A.R., Miranda Filho J.B. (2010). Heterosis. Quantitative Genetics in Maize Breeding.

