# Supplementary material for: Evaluating Maize Hybrids for Yield, Stress Tolerance, and Carotenoid Content: Insights into Breeding for Climate Resilience
Source: Plants (Basel). 2025 Jan 6;14(1):138. doi: 10.3390/plants14010138 (PMC11722938; doi:10.3390/plants14010138)
Supplement: Supplementary file 1 [file plants-14-00138-s001.zip › plants-3378870-supplementary.pdf]

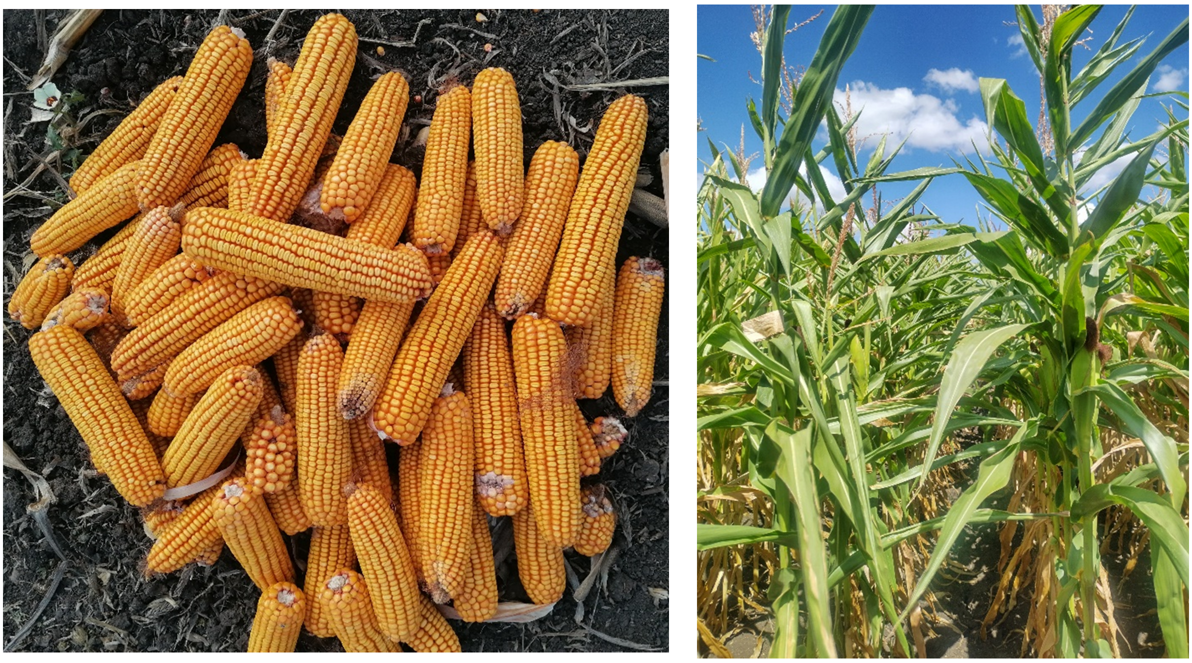

Figure S1. Ears and plants of A447xD302 hybrid

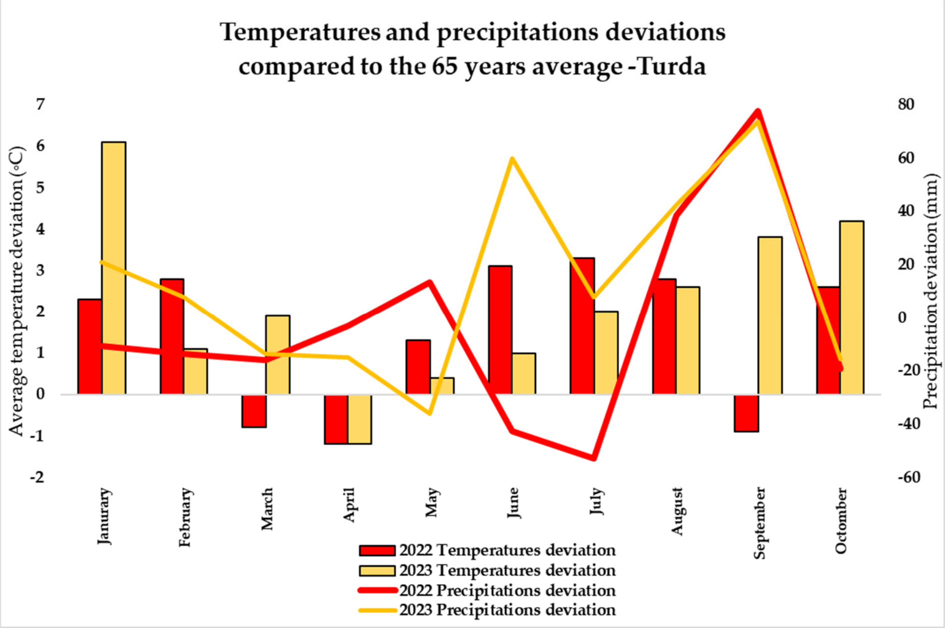

Figure S2. Temperature and precipitations deviations, compared to 65-year average (Turda, 2022-2023)
